# Innovative domestic financing mechanisms for health in Africa: An evidence review

**DOI:** 10.1177/13558196231181081

**Published:** 2023-06-16

**Authors:** Nouria Brikci

**Affiliations:** Research Fellow in health economics, Department of Global Health and Development, 4906 London School of Hygiene & Tropical Medicine, London, UK

**Keywords:** health financing, Africa, innovative

## Abstract

**Objectives:**

This article synthesizes the evidence on what have been called innovative domestic financing mechanisms for health (i.e. any domestic revenue-raising mechanism allowing governments to diversify away from traditional approaches such as general taxation, value-added tax, user fees or any type of health insurance) aimed at increasing fiscal space for health in African countries. The article seeks to answer the following questions: What types of domestic innovative financial mechanisms have been used to finance health care across Africa? How much additional revenue have these innovative financing mechanisms raised? Has the revenue raised through these mechanisms been, or was it meant to be, earmarked for health? What is known about the policy process associated with their design and implementation?

**Methods:**

A systematic review of the published and grey literature was conducted. The review focused on identifying articles providing quantitative information about the additional financial resources generated through innovative domestic financing mechanisms for health care in Africa, and/or qualitative information about the policy process associated with the design or effective implementation of these financing mechanisms.

**Results:**

The search led to an initial list of 4035 articles. Ultimately, 15 studies were selected for narrative analysis. A wide range of study methods were identified, from literature reviews to qualitative and quantitative analysis and case studies. The financing mechanisms implemented or planned for were varied, the most common being taxes on mobile phones, alcohol and money transfers. Few articles documented the revenue that could be raised through these mechanisms. For those that did, the revenue projected to be raised was relatively low, ranging from 0.01% of GDP for alcohol tax alone to 0.49% of GDP if multiple levies were applied. In any case, virtually none of the mechanisms have apparently been implemented. The articles revealed that, prior to implementation, the political acceptability, the readiness of institutions to adapt to the proposed reform and the potential distortionary impact these reforms may have on the targeted industry all require careful consideration. From a design perspective, the fundamental question of earmarking proved complex both politically and administratively, with very few mechanisms actually earmarked, thus questioning whether they could effectively fill part of the health-financing gap. Finally, ensuring that these mechanisms supported the underlying equity objectives of universal health coverage was recognized as important.

**Conclusions:**

Additional research is needed to understand better the potential of innovative domestic revenue generating mechanisms to fill the financing gap for health in Africa and diversify away from more traditional financing approaches. Whilst their revenue potential in absolute terms seems limited, they could represent an avenue for broader tax reforms in support of health. This will require sustained dialogue between Ministries of Health and Ministries of Finance.

## Introduction

The health and fiscal shocks of the COVID-19 pandemic have put into sharp focus the need to strengthen national health systems and the difficulty for governments across the world, and in particular in low- and middle-income countries (LMICs), to invest in them.^1^ Financing of good quality health care across Africa, in particular, remains inadequate: governments allocate too little of their revenues to health, whichever benchmark is used,^2^ and households continue to carry a significant proportion of the financial burden associated with seeking care through out-of-pocket payments.^3^ Increasing fiscal space for health is therefore urgent.

Fiscal space for health can be generated through economic growth, increased prioritization given to health, additional aid allocation, additional borrowing from governments, generating financial savings through greater efficiency in spending existing health resources, and domestic revenue mobilization.^4^ Whilst each of these avenues are important and should not be considered in silo, increased attention has been paid in the past decade to the last of these.^5^ So-called innovative financing mechanisms have generated great enthusiasm for their potential to raise additional domestic resources for health.^6^

The World Health Organization (WHO) defines innovative financing as mechanisms offering avenues for countries with large informal economies to diversify away from well-known approaches that are relatively easy to collect, such as taxes on formal-sector employees and their employers, import or export duties of various types and value-added tax (VAT).^7^ The World Bank Group uses innovative financing as an overarching term that includes any financial approach that enables additional funds generation by utilizing new funding sources or engaging new partners or increasing efficiency by reducing time and service delivery costs.^8^ Innovative domestic financing mechanisms are defined here, using and further specifying the WHO definition, as any domestic financing source that is outside of general taxation, VAT, user fees or any type of health insurance, and from which revenues would be intended to be, or were, allocated to health. This paper investigates whether these domestic innovative financing mechanisms could provide part of the answer to the fiscal crisis facing health systems across the African continent.

## Methods

This paper synthesizes the evidence on domestic innovative financing mechanisms, as defined above, in African countries to answer the following research questions:

• What types of domestic innovative financial mechanisms have been used and documented in relation to health?
• What is known in relation to the additional revenue that these innovative financing mechanisms raise?
• Have these mechanisms been, or were meant to be, earmarked for health?
• What is known about the policy process associated with their design and implementation?

To address these questions, a systematic review was conducted of peer-reviewed and grey literature providing quantitative information about the additional financial resources generated through innovative domestic financing mechanisms for health care in Africa, and/or qualitative information about the policy process associated with their design or effective implementation. A combination of the following search terms was used: ‘domestic’ or ‘national’, and ‘innovative’ or ‘tax*’ or ‘levi*’ (levies being a synonym for taxes) or ‘sin’ (taxes on tobacco and alcohol are sometimes referred to as sin taxes), and ‘health*’, and ‘financing’. Seven databases were systematically searched: Scopus, Pubmed, Global Health, Cochrane Library, Econlit, Embase, Medline. Details on how many articles were obtained from each database can be found in Table S1 in the online supplement. The search was conducted in November 2021.

This search was accompanied with a targeted search of the WHO, OECD, Global Fund and World Bank websites, as these institutions have most published on this topic, but their reports may not be identifiable in standard bibliographic databases. To ensure all highly relevant publications were captured, experts at the WHO and World Bank were contacted to help identify any additional relevant documentation. This process identified an additional four reports.

Articles were selected following the following criteria:

(a) Articles were included if they:

• were published in English or French, the two primary publication languages used in Africa,
• described the policy process associated with designing or implementing innovative domestic financing approaches for health,
• provided quantitative estimates of how much money these mechanisms had the potential of raising, or had raised,
• focused on a single or multiple African countries.

(b) Articles were excluded if they:

• were policy briefings, blogs or material documenting international innovative financing mechanisms. Policy briefings were excluded as they do not undergo systematic peer review processes, which would have limited the quality of evidence;
• were set outside of Africa,
• were not related to financing health,
• did not discuss domestic revenue mobilization,
• did not focus on innovative financing approaches,
• were published prior to 2000,
• were not written in English or French,
• did not provide any quantitative information on revenue raised (or potential for revenue to be raised),
• did not discuss the feasibility of policy implementation.

The same inclusion and exclusion criteria were applied for the WHO, World Bank, Global Fund and OECD targeted searches. When evidence was cited in an article, the references were checked to identify any material that had so far been missed.

For every financing mechanism identified but not yet implemented, a follow-up Google search was undertaken in August 2022 to determine whether the mechanism had subsequently been implemented, and whether the revenue raised had been documented.

## Results

**Figure 1.**
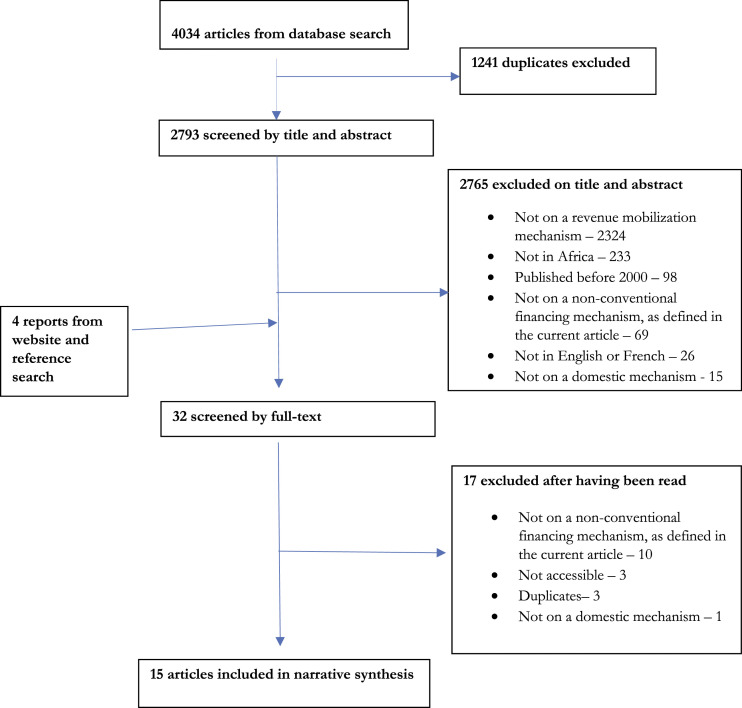
PRISMA diagram detailing the selection of the articles included in this review.

The review search led to an initial list of 4034 articles. Figure 1 details how this original list of articles was ultimately reduced to 15.

These 15 studies were included in a narrative synthesis. The main details of the 15 articles are summarized in Table S2 in the online supplement.

### Type of studies

Ten studies used literature reviews, eight used qualitative methods and five used some form of quantitative analysis (either actual budget data analysis or financial modelling) (see Table 1).

**Table 1.** Research designs of the selected articles.

Article	Literature review	Qualitative methods	Quantitative methods or data
Musango and Aboubacar^9^ (2010)	✓	✓ (Key informant interviews)	✓ (Budget data analysis)
Musango et al.^10^ (2012)	—	✓ (Summary of discussions between Ministers of health and finance)	—
Remme et al.^5^ (2016)	—	—	✓ (Modelling)
Atun et al.^11^ (2016)	✓ (systematic)	—	—
Global fund^12^ (2016)	✓	—	—
Cashin et al. (2017)^13^	✓	✓ (Questionnaire)	✓ (Based on literature review)
Allen^14^ (2017)	—	✓ (Write-up of World Health Organization working group discussion)	—
Elovainio and Evans^15^ (2017)	✓	—	✓ (Based on literature review)
Barroy et al.^16^ (2018)	✓	—	✓ (Cross-country analysis of cross-sectional data)
Chansa et al.^17^ (2018)	—	✓ (Delphi technique)	✓ (Modelling)
Doherty^18^ (2019)	✓	—	✓ (Budget data analysis)
Zakumumpa et al. (2019)^19^	✓	✓ (Key informant interviews)	—
Mathauer et al.^20^ (2019)	✓	✓	✓ (Modelling)
Ifeagwu et al.^21^ (2021)	✓	—	—
Laar et al. (2021)^22^	—	✓ (Key informant interviews)	—

### Range of innovative resource mobilization mechanisms

The types of mechanisms implemented, or considered for implementation, were varied. The most common were taxes on mobile phones (10 articles discussed taxes on mobile phone usage or mobile operators in Gabon, Ghana, Republic of Congo, Senegal, Benin, Mali, Togo, Tanzania, Mozambique and Uganda),^9,12,13,14,15,16,18,20,21,22^alcohol (nine articles analyzed this tax across 14 sub-Saharan African countries including Mali, Benin, Togo, Tanzania, Mozambique, South Africa, Botswana, Malawi, Nigeria and Eswatini),^5,10,11,12,13,14,18,20,21^ eight discussed taxing money transfers (particularly diaspora bonds and remittances, to and from other countries in Gabon, Benin, Mali, Tanzania, Mozambique and Togo),^9,10,12,13,16,20,21,22^ and another eight articles considered taxing tobacco products (in countries such as Egypt, Ivory Coast, Djibouti and Ethiopia).^10,13,14,15,16,18,19,21^ Five articles looked at levies on natural resource extraction in Botswana, Mali, Mozambique, Togo and Ghana,^15,18,20,21,22^ another five looked at soft and sugar-sweetened beverages taxes in Uganda, in particular.^11,13,14,18,19^ Four looked at airline levies or taxes on the tourism industry in Benin, Cameroon, Congo, Madagascar, Mali, Mauritius, Mozambique, Togo, Tanzania and Niger.^11,12,20,21^ Three articles considered levies on fuel (storage), tax on motor vehicle insurance in Malawi and on cars and other vehicles in Mozambique.^17,18,20^ Finally, two examined lotteries^16,20^ and two at profitable industries, such as the banking sector,^18,21^ whilst one article mentioned the possibility of taxing bottled water in Uganda.^11^

### Revenue potential

The evidence on the revenue that could be raised through these mechanisms was limited, with scarce documentation either of their potential or actual revenue raised. Of the six quantitative analyses included, three documented actual revenue raised in Gabon, Egypt, Tanzania and Uganda^9,16,18^ and three used various modelling techniques to project potential revenue streams.^5,17,20^ Two literature reviews gathered additional quantitative analysis.^13,15^

The revenue projected to be raised by these mechanisms was relatively low, ranging from 0.01% of Gross Domestic Product (GDP) for alcohol tax alone,^5^ for example, to 0.49% of GDP if multiple levies were applied.^12^ As a share of general government health expenditure (GGHE), however, these sources could represent a substantial addition - up to nearly 14% of GGHE for mobile phone levies,^15^ and up to 43% of GGHE if multiple levies were applied.^20^ This upper limit was the case of Benin, where taxes on five classes of goods and services simultaneously were considered – alcoholic drinks, aeroplane tickets, mobile communications, financial transactions and the national lottery. The implementation of these five taxes in different sectors was to some extent unrealistic. However, all these figures should be treated with caution, as they are focused mainly on modelling exercises conducted in advance of any implementation of the tax rather than actual data gathered from implemented financing approaches. Further details on potential revenue raised are given in Table S3 in the online supplement.

Few of the mechanisms discussed in the selected studies have apparently been implemented. Subsequent Google searches (conducted in August 2022) to establish whether proposed reforms had materialized suggest that of the taxes proposed, only one country had done so. Botswana introduced a tax on alcohol, of which 10% went to health (Table 2).

**Table 2.** Implementation of the innovative domestic funding mechanisms, as at August 2022.

Country	Nature of innovative funding mechanism	Mechanism implemented?
Benin	Taxes on alcoholic drinks, aeroplane tickets, mobile telephones, financial transactions and national lottery	Unclear. No information found
Botswana	Alcohol tax	Yes. Levy introduced, 10% of which goes to health
Eswatini	Alcohol tax	Unclear. No information found
Malawi	Alcohol tax	No. Tax on alcohol was decreased – no mention of allocation for health
	Increase tax from existing fuel levies (storage and major electrical infrastructure). New tax on motor vehicle insurance	Unclear. No information found
Mali	Taxes on alcoholic drinks, aeroplane tickets, mobile and fixed telephones, financial transactions and extractive industries	Unclear. No information found
Mozambique	Taxes or levies on aeroplane tickets, phone calls, alcoholic drinks, tourism services, financial transactions, lottery tickets, vehicles and the extractive industries	No. However, reform being prepared
Nigeria	Alcohol tax	No. Taxes on alcohol and sugar-sweetened beverages in discussion but no mention of allocation for health
South Africa	Alcohol tax	No. In 2021 increase in tax on alcohol and tobacco products, but no earmarking for health
Tanzania	Remittances levy, airtime levy, alcohol levy and airline levy	No. However, pilot study looking at acceptability of earmarked marginal levy for tobacco, alcohol, sweets/soft drinks and fuel found public support to be overwhelmingly high, at more than 90%
Togo	Taxes on alcoholic drinks, aeroplane tickets, mobile and fixed telephones, financial transactions and extractive industries	No.

### Political acceptability

Taxation was recognized as a political reform, even more so if introduced to prioritize a specific sector. This was the most recurrent theme across the articles selected.^10,12,13,15,16,18,20^ As a result, at the stage of identifying the mechanism to introduce financing, a key task identified was engaging heads of state and parliamentarians over and above the various ministries affected by the potential reform (Ministries of Health and Ministries of Finance, for example).^10,12,13^ This was the case, for example, in Botswana where political leadership by the president was crucial the success of the reform.^12^

The competing interests of central ministries may also create political resistance at central level. For example, in Togo and Benin a new levy on the tourism industry was supported by the Ministry of Health, as the tax was earmarked for health, but was resisted by the Ministry of Trade, which saw the tax as anti-business.^20^ If the interests of autonomous municipalities or districts in decentralized settings are ignored, this can also lead to resistance that hampers reforms, as was the case in Mozambique.^20^ The articles also noted that political acceptability depended on the object of the tax, with greater support for taxes on harmful products for health.^10,13,18^

According to the selected papers, it was also important to understand the full range of institutional reforms needed to implement the taxes. These reforms could be substantial and time consuming,^5^ and depended on whether mechanisms to collect these taxes already existed, whether technical capacity to collect these taxes existed or needed to be built, and whether new laws would be required to enact these mechanisms.^20^ In Botswana, for example, the new tax was supported by legislative reforms (amendments to the Road Traffic Act).^12^

### Industrial acceptability

Several articles noted the importance of involving the targeted industry. In Gabon, for example, mobile phone companies were not consulted about the implementation of a new tax on mobile phones. They learnt about the new levy on their revenue for health through the press.^9^ Such lack of consultation can increase the chances of the targeted industry actively resisting the proposal.

The power of the industries affected by the new taxes also affected whether these would eventually be implemented. In Benin, for example, taxes on the extractive industries were resisted by the country’s large extractive companies, and eventually deemed infeasible.^20^ In Malawi, taxation on tobacco was deemed undesirable by the government, as tobacco, referred as ‘green gold’, was the key export commodity of the country, and involved a large proportion of agricultural producers.^17^ In the case of taxation on mobile phone usage, there were concerns this tax would have a detrimental impact on the promising growth of mobile banking in Africa.^15^

### Design considerations

The question of whether, and how, to earmark the additional revenues for health was a key design feature identified in the literature. All of the innovative mechanisms were expected to be, or were, earmarked for health or HIV, although two proposed innovative mechanisms for health but did not specify whether earmarking would take place.^11,16^ Earmarking was highlighted as a complex issue. Whilst it might allow the financing mechanism to bypass annual budget negotiations and ensure a protected revenue stream for health, it might also decrease the flexibility of budget allocation across sectors, hence reducing the allocative efficiency of public finance.^13^ This might hamper the ability of Ministries of Finance to implement stabilization policies in times of economic turmoil,^13^ and create tension between Ministries of Health and Ministries of Finance.^10^

Earmarking revenue can be even more complicated in decentralized settings, such as Mozambique, where autonomous municipalities might not approve of the centrally driven and sector-specific prioritization inherent in earmarking.^20^ Furthermore, whereas earmarking of revenues for health might be assumed to equate to additional revenues for health, this was not necessarily the case. The introduction of an earmarked financing mechanism could be offset by a reduction in health expenditure in areas that are not part of the earmarking. This happened in Ghana and Gabon,^10,13,16^ and is known as the fungibility of resources. In fact, what evidence there is suggests that even when these innovative mechanisms are introduced, the additional resources that are provided to the health sector are either zero or short-lived due to the fungibility of resources at budget level.^13^

Earmarking was less problematic when health and finance authorities had aligned objectives^13^ or when the earmarking was identified as supporting a politically more acceptable cause.^10^

### Equity impact

Five articles raised the issue of the potentially regressive nature of these mechanisms.^13,15,17,18,20^ In particular, the negative impact of taxes on tobacco, financial remittances and alcohol – all used disproportionately highly by poorer segments of the population – were mentioned.^20^ This potential regressivity in revenue-raising could be counterbalanced by relative progressivity in spending if the poorest segments of the population were targeted for increased health spending.^18^

It was also noted that any revenue-raising approach that contributed to some form of pre-payment away from out-of-pocket payments could be deemed as improving the progressive nature of the health-financing mechanism overall.^18^

## Discussion

Our systematic review found that the available literature on innovative financing mechanisms in Africa was limited, although the suggested products or industries to be taxed were varied. The potential revenue that could be raised through these mechanisms was low when compared to GDP, but could be more substantial when compared to GGHE. The evidence base for this, however, was limited and to some extent unrealistic, as the upper bound (the case of Benin) represented the implementation of up to five new taxes across five different sectors.

Certain policy factors – such as political acceptability, the potential distortionary impact these reforms may have on the targeted industry and the readiness of institutions to adapt to the proposed reform – were identified as being key to consider prior to implementation. This would suggest that tax reforms may be more successful if built on existing systems rather than relying on the creation of new institutions. From a design perspective, the fundamental question of earmarking proved complex, both politically and administratively. Indeed, despite earmarking, the additional resources that are generated for the health sector would appear to be either zero or short-lived. Finally, ensuring that these mechanisms supported the policy’s underlying equity objectives was recognized as important.

### Further research

Despite the importance of identifying additional domestic resource mobilization avenues, much still needs to be understood about the potential and application of domestic innovative financing mechanisms and how to diversify away from the traditional tax approaches used across LMICs. In particular, few studies have looked at the implementation challenges of such reforms (e.g. political acceptability and need for administrative reforms), and how to overcome them. Further research in this area would be essential to fully understand the potential of these mechanisms. This is particularly the case given virtually none of the mechanisms identified in this review have apparently been implemented.

Furthermore, as demonstrated by this review, many avenues to diversify the tax base in LMICs could be further explored, although the political nature of taxation may limit what is feasible. The health literature could engage more systematically with the research and evidence on taxation more broadly.^23^ This could go beyond innovative financing mechanisms to review, for example, the extent to which property taxes could yield additional revenue for health.^24^

The impact on equity of innovative mechanisms should also be more fully investigated. Whilst the potential regressive nature in the short term of health taxes has been noted, this could be offset by a decrease in consumption of harmful products in the medium to long term, particularly for poorer households, and an improvement in health outcomes, or at least a reduction in negative health impact.

There is limited discussion on the desirability of earmarking, and what would make earmarking policies successful. Research into earmarking for health, which is inextricably linked to public finance management systems,^25^ is key, as the ultimate intention of these mechanisms is to provide additional resources for health. Research could focus on: (1) better understanding the contextual characteristics that would ensure translation of earmarked innovative financing mechanisms into additional revenue for the health budget formulation stage, and (2) how to ensure that these resources are protected for health throughout the budget execution stage.

Finally, a better understanding is necessary of how to design and implement the mechanisms to achieve the desired impact on revenue, as the evidence base on the policy factors facilitating their success is still limited.

### Policy implications

The need to increase domestic public resources for health across LMICs in Africa is unequivocal.^2^ The health policy debate has moved away from taxes or levies on products generally, and focuses more specifically on products that have a negative public health impact such as alcohol, tobacco, sugar-sweetened beverages or fossil fuels.^26^ The focus on these mechanisms is partly related to their ‘pro-health agenda’, that is, their positive impact on health outcomes, increasing their political acceptability^1^ and making advocating for them easier with both Ministries of Health and Ministries of Finance.^13^

Whilst the revenue-raising potential of innovative financing mechanisms is not a panacea, they can still form part of the solution. In the case of health taxes, most LMICs do not sufficiently tax products that are harmful to health.^27^ It may therefore be possible to start with these innovative financing mechanisms, given their greater political acceptability, and use them as a catalyst for greater dialogue between Ministries of Health and Ministries of Finance. These mechanisms could generate momentum for broader tax reforms, which remain the most promising pathway towards universal health coverage.^23^ The role of industries, at global and country levels, in resisting taxes on the products they produce and sell, such as tobacco and alcohol, should also be further analyzed.

The question of additionality and fungibility of the resources raised through innovative financing mechanisms should be taken seriously by Ministries of Health, and their destination and use should be closely scrutinized, in close collaboration with Ministries of Finance. This implies that Ministries of Health will need to fully engage with existing public finance management processes, push for the additional resources raised through these mechanisms to be visibly allocated to health at budget formulation stage, and follow their allocation at budget execution stage. This may require capacity building of Ministry of Health staff at central and sub-national levels.

Beyond these technical considerations, and the need for capacity building of Ministries of Health, any taxation reform will need to fit within the social, economic and political conditions of the country, particularly as compliance with taxation is closely related to level of public trust in government policy decisions, and the strength of the social contract between taxpayers and decision-makers.^28^ Identifying windows of opportunity – such as upcoming elections, periods of economic growth, or even a health crisis such as Ebola or COVID (which have highlighted the fundamental importance of well-functioning health systems) – could be a first step to garner public support for increasing an existing tax or introducing a new one.

The emphasis at the international level on supporting additional taxation in LMICs, including across Africa, has been focused on VAT and income tax, and to a lesser extent on the innovative taxes described in this article.^29^ Renewed support from global agencies for broader taxation reform is urgently needed. More broadly, this focus on innovative financing mechanisms should not detract from the importance of greater prioritization of health by governments.

## Limitations

There are three main limitations in this study. First, there is the difficulty of defining innovative financing. Whilst our definition was based on the WHO and World Bank’s initial interpretations of the term, it remains debatable as to whether increasing a tax rate is truly innovative. Nevertheless, the fundamental message of the potential for diversification through less traditional funding approaches remains valid and guided the selection of articles.

Second, there is the issue of which literature we chose to include. As we considered only literature written in English and French, this may have led to the omission of research and analysis undertaken and published in other languages. However, the fact that this review is focused on Africa, where French and English are the dominant publication languages, hopefully means that little has been missed. The focus on specific grey literature, at the expense of government or consultancy reports, may also have led to the omission of additional evidence.

Third, no quality assessment of the literature selected was applied. This was because study designs were varied and included some types of study for which standard guidelines are not available (e.g. financial modelling studies). This may mean that evidence of a lesser value has been included. Although this meant a broader range of policy considerations was included than might otherwise have been the case, it does mean that evidence of a lesser quality may have been included.

## Conclusions

Despite the limited additional revenue that innovative domestic financing mechanisms raise, and the lack of clarity as to whether they result in a net increase in health spending, Ministries of Health and Ministries of Finance must discuss such mechanisms more fully if Africa’s health sector financial crisis is to be addressed. Additional research should focus on better understanding the design choices made to date and their impact on financing health, as well as on how to design these mechanisms in such a way that they are more likely to be accepted and lead to an increased overall fiscal envelope available for health.

## Supplemental Material

Supplemental Material - Innovative domestic financing mechanisms for health in Africa: An evidence review Supplemental Material for Innovative domestic financing mechanisms for health in Africa: an evidence review by Nouria Brikci in Journal of Health Services Research & Policy
