# Supplementary material for: Innovative domestic financing mechanisms for health in Africa: An evidence review
Source: J Health Serv Res Policy. 2023 Jun 16;29(2):132–40. doi: 10.1177/13558196231181081 (PMC10910821; doi:10.1177/13558196231181081)
Supplement: Supplemental Material - Innovative domestic financing mechanisms for health in Africa: An evidence review [file sj-pdf-1-hsr-10.1177_13558196231181081.pdf]

Journal of Health Services Research & Policy

Innovative domestic financing mechanisms for health in Africa: an e

Brikci N

ONLINE SUPPLEMENT

Table S1: search results

**Database**	**Articles identified**
Ovid-Medline	691
Scopus	1,033
Pubmed	1,339
Global Health	212
Cochrane	9
EconLit	199
Embase	551
Total	4,034

Table S2: Summary of the 15 selected articles

**Full reference**	**Year of publication**	**Study type and design**	**Geographical focus**
Musango L, Aboubacar I. Assurance Maladie Obligatoire au Gabon : un atout pour le bien être de la population, Background Paper, No 16 In: WHO. World Health Report 2010 - Health financing: the path to UHC. Geneva: WHO; 2010	2010	Grey literature review, KIIs and budget data analysis.	Gabon
Musango L, Orem JN,, Elovainio R, et al. Moving from ideas to action - developing health financing systems towards universal coverage in Africa *BMC Int Health Hum Rights* 2012; 12, 30	2012	Write up of panel discussions organized on health financing bringing together Ministers of health and Ministers of finance with the objective of creating a discussion space where the different perspectives on key issues and needed actions could meet.	Africa

Remme M, Siapka M, Sterck O, et al. Financing the HIV response in sub-Saharan Africa from domestic sources: Moving beyond a normative approach *Soc Sci Med*. 2016;169:66-76	2016	Modelling exercise.	14 countries in SSA with highest prevalence of HIV
Atun R, Silva S, Ncube M, et al. Innovative financing for HIV response in sub-Saharan Africa. J Glob Health 2016 Jun;6(1):010407	2016	Systematic literature review	sub-Saharan Africa (SSA)
Cashin C, Sparkes S, Bloom D Earmarking for health: From theory to practice. Geneva;	2017	Literature review and case studies	Global

Global Fund. Innovative and domestic financing for health in Africa - Documenting good practices and lessons learnt; Geneva; Global Fund; 2016.	2016	Case studies using document reviews	Africa
Allen LN. Financing national non-communicable disease responses. Glob Health Action	2017	Write up of findings of the WHO Global Coordination Mechanism on NCDs financing	LMICs
Elovainio R, David E. Raising more domestic money for health: prospects for low- and middle-income countries, *Health Econ Policy Law* 2017; 12, 139–157	2017	Not specified but seems to present evidence from a review of the literature	LMICs
Barroy H, Sparkes S, Dale E, et al. Can Low- and Middle-Income Countries Increase Domestic Fiscal Space for Health: A Mixed-Methods Approach to Assess Possible Sources of Expansion, Health Syst Reform,2018; 4:3, 214-226	2018	Mixed-methods approach, beginning with a literature review then followed by a quantitative, cross-country analysis of cross-sectional data.	LMICs

Chansa C, Mwase T, Matsebula TC, et al. Fresh Money for Health? The (False?) Promise of "Innovative Financing" for Health in Malawi Health Syst Reform 2018;4:324-335	2018	The two-step review methodology uses (1) the Delphi (qualitative) forecasting technique to assess the feasibility of generating additional tax revenue based on expert opinion and document reviews and (2) GDP-based effective tax rate (ETR) quantitative forecasting method to quantify the amount of revenue that could be generated from existing and new sources.	Malawi
Doherty J. Critical assessment of different health financing options in east and southern African countries - Discussion paper 119. Harare; EQUINET; 2019.	2019	Discussion paper - no method outlined	Eastern and Southern Africa (ESA)
Zakumumpa H, Bennett S, Ssengooba F. Leveraging the lessons learned from financing HIV programs to advance the universal health coverage (UHC) agenda in the East African Community. Glob Health Res Policy. 2019 Sep 13;4:27.	2019	Literature review of studies reporting financing mechanisms for HIV programs between 2004 and 2014.and a mixed-methods study	East African Countries

Mathauer I, Koch K, Zita S, et al. Revenue-raising potential for universal health coverage in Benin, Mali, Mozambique and Togo. Bull World Health Organ 2019; 97, 620 - 630.	2019	Country case studies using combination of quantitative modelling and qualitative methods	Mozambique<br>Togo<br>Benin<br>Mali
Ifeagwu SC, Yang JC, Parkes-Ratanshi R, et al. Health financing for universal health coverage in Sub-Saharan Africa: a systematic review. Glob Health Res Policy 2021; 6, 8	2021	Systematic literature review	SSA
Laar AS, Asare M. Dalinjong PA. What alternative and innovative domestic methods of healthcare financing can be explored to fix the current claims reimbursement challenges by the National Health Insurance Scheme of Ghana? Perspectives of health managers. Cost Eff Resour Alloc 2021;19, 69	2021	A qualitative study using in-depth interviews with 16 health facility managers were held.	Ghana

Table S3: Revenue potential as % of GDP and GGHE

Country	Taxes on	Article documenting a proposal or implemented mechanism?	Revenue raised as % of GDP
**Gabon**	Mobile phone	Implemented	0.20%
	Transfers	Implemented	0.07%
**Malawi**	Alcohol tax	Proposal	0.19%
**Nigeria**	Alcohol tax	Proposal	0.01%
**Eswatini**	Alcohol tax	Proposal	0.09%
**Botswana**	Alcohol tax	Proposal	0.09%
	Alcohol tax	Implemented	0.06%
**South Africa**	Alcohol tax	Proposal	0.03%
**Tanzania**	Remittances Levy, Airtime Levy, Alcohol Levy and Airline Levy	Proposal	0.49%
**Egypt**	Tobacco tax	Implemented	0.03%
**LMICs**	Public health taxes (Tobacco tax, Mobile phone levy, Tax on remittances, Tax on health specific lotteries)	Some implemented, others no. Which was not specified	0.02% to 0.05%

**Malawi**	Increase tax from existing fuel levies (storage and MAREP)<br>New tax on motor vehicle insurance.	Proposal	between 0.19% and 0.21% of GDP
**Mozambique (proposed scenario)**	Taxes or levies on airplane tickets, phone calls, alcoholic drinks, tourism services, financial transactions, lottery tickets, vehicles and the extractive industries.	Proposal	0.20%
**Togo (proposed scenario)**	Taxes on alcoholic drinks, airplane tickets, telephone (mobile and fixed), financial transactions and extractive industries.	Proposal	0.24%
**Benin**	Taxes on alcoholic drinks, airplane tickets, telephone (mobile), financial transactions and national lottery	Proposal	0.28%
**Mali (proposed scenario)**	Taxes on alcoholic drinks, airplane tickets, telephone (mobile and fixed), financial transactions and extractive industries.	Proposal	0.15%

Notes: GDP and GGHE data for the relevant years were obtained from the WHO Global Health Expenditure database https://apps.who.int/nha/database/ViewData/Indicators/en – Exchange rates for the relevant years were obtained from the IMF database using the domestic currency per US dollar, period average as per https://data.imf.org/regular.aspx?key=61545850

Under Review

evidence review

Non-conventional revenue mobilisation mechanism discussed	Earmarked (or intended to be)	Operational
Mobile phone levy<br>Financial transfers abroad	Yes	Yes
Levies on remittances and Foreign Direct Investment (FDI)<br>Levies on tobacco and alcohol	Yes	No

iew

Increased revenues from increase in excise tax on alcohol (beer)	Yes	No
Other HIV trust funds planned in Kenya (multiple sources), Tanzania (unknown source), Uganda (source of revenue are taxes from alcohol, soft drinks and bottled	Yes	No
Several countries have introduced their own versions of airline levies, including Cameroon, Congo, Madagascar, Mali, Mauritius and Niger, and is under consideration in Benin, Burkina Faso, Central African Republic, Gabon, Guinea, Kenya, Liberia, Namibia, Senegal, São Tomé	Not known	Yes
Tax on alcohol<br>Tax on sugar-sweetened beverages<br>Tobacco tax	Yes (fully or partially)	Yes overall

Alcohol levy in Botswana - The alcohol levy was introduced in November 2008 at a rate of 30% through the Levy on Alcoholic Beverages Fund Order.The levy was subsequently raised in 2010 to 40%, then 45% in 2013 and 55% in January 2015. Tanzania - Remittances Levy, Airtime Levy, Alcohol Levy and Airline Levy as well as to implement private sector mainstreaming	**Botswana** - The policies and activities of the levy managed by the Alcohol Levy Implementation Committee housed in the Ministry of Health.- Funds collected from this tax are used to support public education and rehabilitation programmes as well as law enforcement measures to combat alcohol abuse.**Tanzania** - revenue intended for health	Botswana - yes Tanzania - no
**Compulsory levies or taxes -** An increasing number of countries are applying 'sin taxes' to unhealthy products and using the	Yes	Yes
Natural resource levy/ taxation for health<br>Taxes on mobile phone use or turnover of mobile phone companies	Yes	Yes
Tobacco tax	Yes in Egypt (the 10 piastre share,	Yes
Tobacco tax<br>Mobile phone levy<br>Tax on remittances<br>Tax on health specific lotteries	Not known	Not know

iew

Increase tax from existing fuel levies (storage and MAREP)<br>New tax on motor vehicle insurance.	Meant to create additional fiscal space for health	No
Taxes on harmful substances (in Moz beverages inc beer, tobacco, sugar - in Botswana intend to introduce - in South Africa: recently introduced sugar tax but no earmarking)<br>Taxes on mobile phones (Uganda, in Zimbabwe there is a 3% earmarked levy for health which was recently imposed on mobile airtime data),<br>Taxes on motor vehicles (in Zimbabwe for health - in South Africa fuel levy for the Road Accident Fund) ,<br>Taxes on mining sector<br>Taxes on profits (Zambia)	yes but not in SA, Uganda, Zambia	yes mostly (SA, Moz, Uganda, Zimbabwe, Zambia) - not yet in Botswana
Tax on tobacco products and soft drinks<br>In 2008, Ivory Coast introduced a 'solidarity' tax on tobacco products which goes into the National AIDS Fund (FNLS) for funding that country's national HIV response.<br>Uganda enacted a law establishing the national AIDS Trust Fund (ATF) in July 2014 . The AIDS Trust Fund will be supported by a tax on soft drinks in Uganda and presents the promise of increasing country ownership of HIV programs	yes for HIV/ AIDS response	Yes

iew

Taxes or levies on airplane tickets, phone calls, alcoholic drinks, tourism services, financial transactions, lottery tickets, vehicles and the extractive industries.	yes	no
Alcohol, tobacco (Khat in Egypt), air travel, mobile phone call tariffs, raising diaspora bonds, taxation of profitable sectors, such as the banking or petroleum industry.	yes	yes
Natural resources such as crude oil and gas, gold, bauxite, cocoa, mobile money transfers, airtime	Yes	No

iew

iew

**Quantitative information on additional revenue generated (effectively or potentially) through financing mechanism**
Mobile phone companies provide 10% of their revenue before tax from mobile phone business to the state to finance the national health insurance fund (CNAMGS)<br>All money transfers abroad outside economic zone of 1.5% of revenue before tax to CNAMGS.<br>The funds collected for the CNAMGS in 2009 are estimated at 12.2bn FCFA from the mobile phone companies, and 4bn FCFA from transfers.
No

Per year across the 14 countries, US$171 million from increased alcohol taxation (average of US$12.2M a year per country)<br>In Africa (in 2014USD):<br>Malawi - USD11.28M per year 2014-2018<br>Nigeria - USD41.18M per year 2014-2018<br>EsWatini - USD4.06M per year 2014-2018<br>Botswana - USD14.38M per year 2014-2018<br>South Africa - USD99.86M per year 2014-2018
No data provided as had not been operationalised yet
No data provided
**Gabon** - According to budget figures, the initially increased funds for the health sector as a result of the new earmarked tax on mobile phone company revenues have been washed out, with overall health sector budget resources returning to the levels

Botswana - Since the introduction of the alcohol levy in 2008, US$79 million has been collected (by 2016 so over 8 years or about USD9.8M per year)
Tanzania - proposal of five levies (Remittances Levy, Airtime Levy, Alcohol Levy and Airline Levy as well as to implement private sector mainstreaming) the total funding that this would generate would amount to USD $246 million per year.

None
Botswana - taxation on natural resources (diamonds)- substantial resources raised - no data<br>Gabon, Ghana, Senegal, Republic of Congo, Uganda - telephone services<br>Uganda - tax revenue from telecommunication companies amounted to $3.57 billion in 2008, representing 9.5% of the country's total tax revenue. However this did not specify how much came from innovative mechanism, and includes VAT and other taxes on telecoms.<br>Gabon - Gabon implemented a tax of 10% on the turnover of mobile phone companies. The revenue is earmarked for the national health insurance fund and covers the membership of people who cannot afford to contribute . In 2009 the government collected 12 billion francs CFA, or US$25 million, from it<br>Togo - In early 2015, Togo adopted a tax (25 francs CFA or 4 US¢) on incoming international calls; the revenue from this tax is earmarked for the national health insurance mechanism
Egypt - For fiscal year 2013/2014, the projected annual revenue from combined tobacco taxes was EGP 32 billion ($4.2 billion) although this is combined tobacco
When quantified, the potential gains from tobacco tax earmarking comprise between 0.02% and 0.05% of GDP.<br><br>Other types of earmarked taxes, including mobile phone levies, remittances, and health-specific lotteries, also appear to provide limited additional resources, ranging from 1% to 2.3% of actual spending on health

Assuming that all three levies are introduced, 5.4 million USD would be raised on average per year over the period 2016–2017 to 2021–2022 under the low scenario. Under the high scenario, the amount of revenue generated would be 11.6 million USD on average per year over the period 2016–2017 to 2021–2022.
In Uganda - tax for health on mobile phone use and handset sales that raises 9.5% of total tax revenue, but not specifically targeted at the health sector<br>In Zambia - in 2003, medical levy of 1% was imposed on all gross interest earned on savings and a variety of financial instruments used for AIDS treatment. This raised around $2 million annually but was abolished in 2013
No

Yes - Estimated additional revenues through new revenue-raising mechanisms ranged from 0.47–1.62% as a share of general government expenditure in the four countries for the low level scenario, and 0.47–1.62% across the four countries, or 0.52–2.88% for the high-scenario case.<br>As a share of GDP, between 0.09% to 0.74%
no
No

**Discussion of policy process associated with development or implementation of mechanism**
Mobile operators were not consulted prior to the implementation of the tax and learnt about it via the press. They argue that the tax does not allow them to 'breathe' and authors suspect that the cost of paying this tax is assigned to the consumer rather than absorbed by the mobile companies.
Health advocates often push for "sin taxes" since they have a direct public health effect through the reduction of consumptions of these products; MoF supportive as represent additional revenue. The earmarking of these taxes is contentious with MoF.<br>Focus on financial management and on transparency and accountability of these mechanisms and schemes– at all levels - are prerequisites.<br>Many of the decisions of resource allocation are political; making the case for health should also target political actors<br>Health financing reforms, actions and interventions cannot happen in a purposeful way without robust, context specific evidence.<br>The unsatisfactory state of health financing mainly attributed to lack of clear vision and evidence based plans and strategies.

Recognise that institutional reforms required would be substantial and time-consuming but does not elaborate on this point.<br>Any earmarking may reduce fiscal flexibility and allocative efficiency in public finance more broadly and may not be acceptable to ministries of finance. Moreover, it is quite likely that increased revenues from HIV-earmarked sources may in practice be accompanied by a reduction in allocations from general government revenue to HIV
**No**
**Article discusses earmarking in depth:**<br>Can be revenue or expenditure earmarking<br>Earmarking has become part of the global discussion on domestic resource mobilization for health,

**Botswana** - - High-level political commitment and visionary leadership was key in successful implementation.- Extensive research prior to implementation including a report on the impact of the alcohol levy, comparison to other interventions as well as consistent reviews to monitor its impact were undertaken.- Legislative revision such as amendment of the Road Traffic Act, separating the Trade and the Liquor Act in to two Acts and drafting of the Traditional Beer Regulations provided a legal framework for the implementation of the new measures<br>**Tanzania** - no analysis of technical or political feasibility
No
**Earmarking** - MoFs do not favor hypothecation where taxes are earmarked for health as it reduces flexibility to allocate funds to all sectors as needed. Tax increases through earmarked taxation may be more motivating for tax payers as they can directly understand the cause for which the tax is used.<br>**Politics** - Considerations noted as important before introducing innovative taxes: · Adequacy and stability of the revenue raised; · efficiency: that is, that it does not introduce major imbalances in the economy and/or distortions in behavior; equity and impact on the poor; ease and costs of collection; political acceptability. No further details however provided as to what these entail. Concerns that taxes are distortionary – for example, taxes on mobile phones could reduce their potential to be used for 'good' purposes such as the transmission of health data. A general concern is also that some forms of taxes are regressive.
Few fiscal space for health studies disentangle potential from general tax policies, and analyze the actual capacity and constraints to effectively mobilize, administer and redistribute general revenues, including for health issues. Studies should also always consider political feasibility and not take for granted the additivity of public health tax revenues to the health sector budget.

Difficult to increase taxes in already existing areas without causing adverse effects on production, trade, and consumption.<br>Perspectives on taxes on increasing taxes on alcohol and tobacco described: consumption of alcohol is low in Malawi, hence revenue would be limited anyhow. Tobacco is called green gold, and is the country's key export commodity, accounting for almost half of the country's total merchandize exports. Also, tobacco production is already highly taxed and politicized, and smuggling of tobacco to neighboring countries is rife.
Taxes on harmful substances such as sugar and tobacco can be an instrument for revenue collection and a negative incentive for consumption of these substances<br>This can make them socio-politically easier to earmark for health spending, as they are understood to have a negative impact on health. For example, Egypt has increased revenues from a tobacco tax. The taxes could, however, also be regressive, as was found with the South African alcohol tax , where poorer people smoked more than the wealthier did.<br>They can also lead to smuggling of poor quality versions of these substances.<br>Author also discussed what it would take to improve taxation rates in ESA: strengthening tax collection capacity, reforming tax policy, introducing innovative financing, earmarking resources<br>Discusses question of earmarking in Zimbabwe and states that overall earmarking can have negative implications such as rigidity in budget allocations, constraints to shifting spending according to changing needs and reduced allocative efficiency.<br>Lists advantages of innovative mechanisms: . (1) in terms of improving equity of system: Can improve financial protection, Will tend to improve progressivity as often directed at taxing the wealthy more effectively, directly or indirectly through use of luxury goods and services. Removing subsidies eg on fuel can to impact positively on equity. Achieve health risk cross-subsidies, provided they offer services according to need. (2) In terms of efficiency of system: increases pooling. may add complexity to the tax collection system, Impact on efficiency generally not known. Removal of fuel subsidies may drive up prices generally, including in the health sector. (3) in terms of improving sstainability of system: . Open new sources of revenue for the health sector. Taxes on the exploitation of natural resources can bring in substantial revenue, but no guarantee that the health sector will benefit; The macro-economic concerns are mixed and/or unclear; There may be political resistance to imposition of new taxes, and the removal of subsidies.
No

yes - looked at political feasibility, financial stability and sustainability, progressivity, administrative efficiency and any other possible effect/ barrier to implementation
no
No but uses examples from other countries as justification: In Gabon, the government imposed 10 % levy on mobile phone use in 2008 to fund for national health insurance system to fnance critical healthcare services especially for the poor population . In 2018, the government of Uganda impose d 0.5% tax on the value of mobile phones and 1% on value of mobile money withdrawals to raise a dditional funds for the health sector . Similarly, raising additional funds to fnance healthcare, the Zi mbabwean government imposed a 3% levy on mobile airtime data. Also, in Kenya, exercise taxes w ere imposed on money trans┐fer services, such as airtime and internet data to fnance healthcare. i n Botswana, the government imposed special taxes on diamonds to fnance healthcare . Also, in An gola, to raise funds through local sources to fnance healthcare, the the government imposed levies on earnings from the export of crude petroleum

**Follow up google search - mechanism implemented? If so, revenue raised documented?**

**In Malawi,** excise tax on malt beer and liquor was decreased in 2019 from 90% to 65% (https://www.mra.mw/news/new-customs-and-excise-tax-measures-in-full-effect)<br>**In Nigeria**, the Nigerian Government signed into a law a policy on 31 December 2021 that mandates the payment of an excise duty of 10 NGN (about US$0.02) per liter on all non-alcoholic and sweetened beverages in the country http://www.populationmedicine.eu/Taxation-on-beverages-in-Nigeria-Impact-and-burden-of-the-new-policy,146129,0,2.html). In March 2022, a circular announced the introduction or increases in Excise Duties rates on Non-alcoholic Beverages, Alcoholic Beverages, Cigarettes, Tobacco-Related Products, and Telecommunication Services. Teh rates of increase were announced per consuption. Not yet a Law however. No mention of allocation for health nor HIV. https://taxaide.com.ng/2022/07/07/nigerias-2022-pro-health-taxes/?utm_source=rss&utm_medium=rss&utm_campaign=nigerias-2022-pro-health-taxes<br>**in Eswatini** - no further information found<br>In **Botswana** - In 2008, the government of Botswana imposed a 30% tax levy on all alcohol products to deal with problematic drinking in the country. The levy was initially set at 70% but was reduced to 30%. https://www.issup.net/knowledge-share/research/2020-08/beer-drinking-and-tax-levy-botswana. Resources from the alcohol levy are distributed as follows, 45% to Ministry of Youth, Sport and Culture, 10% to the Ministry of Health and 45% to the Government Consolidated Fund.(Global Fund, 2016)<br>**In South Africa** - South Africa’s finance minister Wednesday announced in 2021 an increase of 8% in excise duties on alcohol and tobacco products to discourage their consumption and promote good public health. No earmarking for health

**Tanzania** - a tax on mobile money but not for health was introduced then drastically reduced as was controversial https://www.aa.com.tr/en/africa/tanzania-slashes-controversial-mobile-money-levy/2619506. no other introduced tax clearly earmarked for health - tax on fuel levy piloted in 2021 and earmarked for health

no information found on motor tax introduced to finance health
Introduced in Botswana since

**Mozambique** - The Mozambican Ministry of Finance is preparing a reform of the Specific Consumption Tax (ICE),which includes taxes on tobacco, alcohol, sugar-sweetened beverages, cars, and other consumption goods.https://p4h.world/en/news/mozambique-review-health-taxes-view-rising-ncd-prevalence#:~:text=Health%20taxes%20currently%20represent%201.3,of%20the%20Ministry%20of%20Finance.<br>**Togo** - no new tax on tobacco for health https://opendocs.ids.ac.uk/opendocs/bitstream/handle/20.500.12413/17218/Togo_Tobacco_Tax_Factsheet.pdf?sequence=1&isAllowed=y - otehr tax incrases introduced but no information of earmarking for health https://www.lloydsbanktrade.com/en/market-potential/togo/taxes<br>**Benin**- no information found<br>**Mali** - no information found
